# Somatic Mutation of *PIK3CA* (H1047R) Is a Common Driver Mutation Hotspot in Canine Mammary Tumors as Well as Human Breast Cancers

**DOI:** 10.3390/cancers11122006

**Published:** 2019-12-12

**Authors:** Kang-Hoon Lee, Hyeon-Ji Hwang, Hyun Ji Noh, Tae-Jin Shin, Je-Yoel Cho

**Affiliations:** 1 Department of Biochemistry, BK21 Plus and Research Institute for Veterinary Science, School of Veterinary Medicine, Seoul National University, Seoul 08826, Korea; khlee02@snu.ac.kr (K.-H.L.); hhyunji1027@naver.com (H.-J.H.); taejin430@snu.ac.kr (T.-J.S.); 2 Broad Institute of MIT and Harvard, 415 Main Street, Cambridge, MA 02142, USA; hyunjiro@gmail.com

**Keywords:** breast cancer, WES, exome-seq, transcriptome, dog, mammary gland tumor, CMT

## Abstract

Breast cancer is one of the most frequently diagnosed cancers in both women and female dogs. Genome-wide association studies in human breast cancer (HBC) have identified hundreds of genetic variations and somatic driver mutations. However, only a handful of variants have been studied for rare HBC and their associations remain inconclusive. Spontaneous canine mammary tumor (CMT) is a great model for HBC, with clinical similarity. We thus performed whole-exome sequencing in 20 pairs of CMT and normal tissues in dogs. We newly found that *PIK3CA* was the most frequently mutated gene in CMT (45%). Furthermore, canine *PIK3CA* A3140G (H1047R), at what is known as the mutational hotspot of HBC, is also a hotspot in CMT. Targeted sequencing confirmed that 29% of CMTs had the same *PIK3CA* A3140G mutation. Integration of the transcriptome suggests that the *PIK3CA* (H1047R) induced cell metabolism and cell cycle via an increase of *PCK2* and a decrease of *CDKN1B* but had no effect on cell apoptosis. We identified additional significantly mutated genes, including *SCRN1* and *CLHC1*, which have not been reported in HBC. Our study recapitulated some known HBC-associated genes and human cancer signatures in CMT, and identified novel genes that may be relevant to HBC. This study may allow us to better understand both HBC and CMT and lend new insights into the development of biomarkers.

## 1. Introduction

Human breast cancer (HBC) now affects one in four women worldwide [1]. It is well known that women who have a family history of HBC have an increased risk of developing HBC. Some high-penetrance mutations responsible for an increased risk of HBC have been found, such as *BRCA1* and *BRCA2*, *PTEN*, *RAD50*, and *TP53* [2]. Over the last few decades, together with the development of high-throughput sequencing technology, a huge number of genome-wide association studies (GWAS) have identified many genetic loci that are associated with HBC and have been able to explain up to 18% of heritability [3]. However, this percentage will be increased with the consideration of recent GWAS data which revealed that silent mutations (synonymous mutations without amino acid changes) frequently contribute to various cancers [4]. This may suggest that HBC is a very complex disease and non-genetic factors, as well as the heterogeneity of human populations, need to be addressed and overcome to discover missing high-risk genes. Studies comparing matched tumor/normal pairs (T/N) have revealed many probable driver genes for HBC, including *TP53* and *PIK3CA*, that are most frequently mutated [5,6].

Canine mammary tumors (CMTs) are potentially a good natural animal model for HBC due to their biological and pathophysiological similarities, as well as an increased rate of incidence and malignancy in dogs. CMT’s molecular characteristics resemble HBC’s, including the expression of estrogen receptor (ER), progesterone receptor (PR), Ki-67, epidermal growth factor receptor 2 (EGFR), basal cytokeratins 5/6, and p53 mutation [7]. Mixed type CMTs share several histopathological features and risk factors with metaplastic HBC and complex subtype CMTs offer an ideal system to study myoepithelial cells, the second major cell lineage of the mammary gland [8,9]. Genes and variations known as high-risk factors in HBC, such as single nucleotide polymorphisms (SNPs) in *BRCA1*, *BRCA2*, and *ER*, have also been reported to be significantly associated with CMTs [10,11]. In addition, Liu D et al. reported molecular similarities such as copy number variation and epigenetic modification between CMTs and HBC, but they could not show significant somatic mutations in CMTs due to the limitation of small sample size [9]. Interestingly, breed predispositions to various types of cancer have been reported, including sarcomas, mast cell tumors, lymphoma/leukemia, melanoma, and mammary tumors [12]. Specifically, Yaritza Salas et al. reported breed predisposition in specific subtypes of CMTs—Poodle and Schnauzer breeds showed the strongest association with complex and tubular type carcinomas, while Cocker Spaniels showed a higher correspondence with carcinosarcoma and mesenchymal origin tumors [13]. The Maltese breed was mainly associated with epithelial tumors, which are the most common type in HBC [13]. These possibly indicates that specific dog breeds are enriched with risk alleles for specific subtypes of CMT.

The goal of this study is to determine mutation profile in CMTs that may help us to understand better the mutations rare in HBC. In this study, we performed exome sequencing in T/N tissue pairs of CMTs from two canine breeds to explore germline and somatic variations relevant to CMTs.

## 2. Results

### 2.1. Whole Exome Sequencing in CMT Was Performed

**Figure 1:**
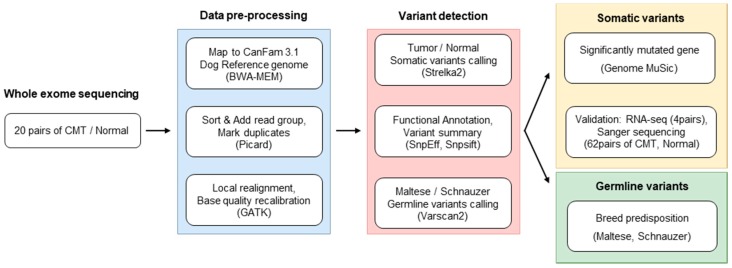
Experimental scheme. The overall procedure of exome sequencing was depicted with major information, such as numbers of specimens, tools, and parameters.

A total of 20 paired CMTs and adjacent normal tissues from small sized dog breeds were subjected to genomic DNA isolation, followed by high-throughput whole exome sequencing (Materials and Methods; Figure 1). The average age was 11.5 years and half were spayed (Table S1). It was unexpected because it is well-known that the risk of a dog developing a mammary tumor is less than 0.5% if they are spayed before the first estrus, while the risk increases to 26% after the second or any estrus. The high frequency of spayed dogs in this study can be explained by the fact that only 2.7% of dogs underwent spaying surgery before 1 year of age, and more than 60% of dogs get spayed after 2 years of age in Korea (Report for Survey on the Recognition and Parenting Status of Pets in 2018) [14,15].

Sequence reads were mapped to the CanFam3.1 dog reference genome, revealing a median depth of coverage of ~102 × in both normal and CMT, and a read depth of at least 30 × in ~95% of the targeted exome region (Table S2). In total, we identified 260,597 germline and 21,448 somatic mutations, which include both synonymous and non-synonymous mutations, with 94,161 germline variants and 1092 somatic variants on average per individual dog (Table S3).

### 2.2. CMT-Enriched Mutation Signatures Are Similar to Pan-Cancer Signatures and Tobacco-Associated Mutational Processes In Humans

**Figure 2:**
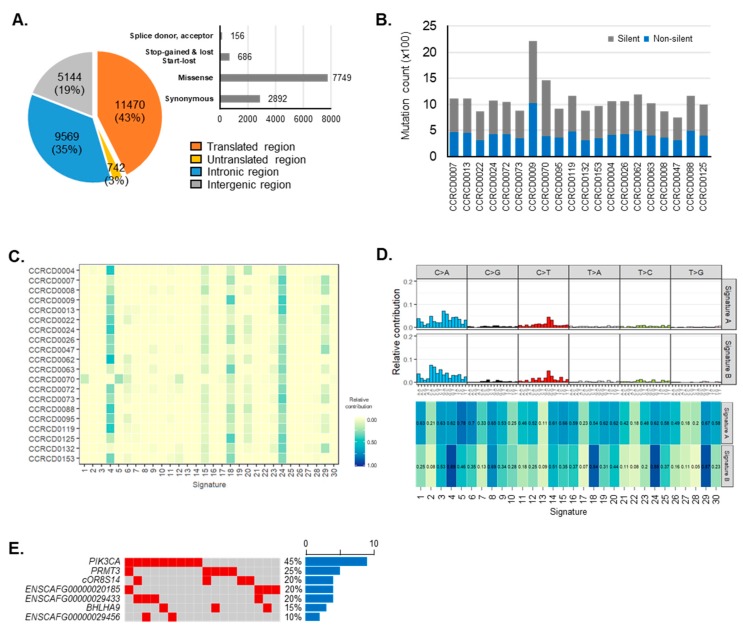
Somatic mutations in canine mammary tumors (CMTs). (**A**) Distribution of somatic mutation in genomic features and specimens. Genomic distribution of somatic mutations was depicted by pie chart and mutations on the transcribed region were departmentalized based on four effects. (**B**) The amount of somatic mutations found in each specimen were separately counted based on silent (gray) and non-silent mutations (blue). (**C**) Mutation profiles in 20 specimens were fit into 30 human cancer signatures. (**D**) De novo signature detection defined two major mutation profiles with corresponding CMT mutational signatures. Signature similarity of two CMT signatures was presented by cosine similarity in 30 human cancer signatures. (**E**) Significantly mutated genes (SMGs) were depicted with frequency across all samples.

We utilized Strelka2 with a default parameter to call somatic variants and identified a total of 21,448 variations including 8582 non-silent mutations. Approximately 43% (11,470) of somatic mutations occurred on exonic regions and these were composed of 7749 missense, 2892 synonymous, 686 stop-gained, stop-lost or start-lost, and 156 splice donor or acceptor mutations. Non-exonic regions were also captured and sequenced for somatic mutations from the introns (35%), intergenic regions (19%), and untranslated regions (3%) (Figure 2A). On average, 1092 somatic mutations were identified from each individual dog (maximum: 2212 mutations in CCRCD0009; minimum: 746 mutations in CCRCD0047). Except for the CCRCD0009 sample, which harbored two times more accumulated somatic mutations, most CMT specimens had average of ~433 non-silent somatic mutations (Figure 2B, Table S4). Since no flaw was found in sequencing data quality, the CCRCD0009 sample was not excluded.

Many association studies have proposed cancer-associated mutational signatures which may occur through different mutational processes [16,17]. In the present study, to identify CMT-enriched mutational signatures, we employed an algorithm recently developed and published by Alexandrov et al. which extracts mutational signatures from catalogues of somatic mutations (http://cancer.sanger.ac.uk/cosmic/signatures) [18,19]. The CMT mutational profiles appeared similar in 96 substitution classifications across 20 specimens (Figure S1A). Overall, transversion base- substitution of cytosine (C) to adenine (A) was the most frequent mutation in CMTs, followed by cytosine (C) to thymine (T) transition (Figure S1B). Although the correlation level was not high, CMT mutational profiles in individual dogs tended to have frequent similarity with certain types of human cancer signatures, particularly Signature 4 (mean correlation: 0.8432, range: 0.6784–0.8913, SD: 0.044) and Signature 24 (mean correlation: 0.8367, range: 0.6625–0.8952, SD: 0.047). Signature 4 has been found in various other cancer types, but not in breast cancer and it is likely due to tobacco mutagens, whereas Signature 24 has been found in cancer triggered by certain toxin exposures (Figure 2C). To determine the CMT-enriched mutational signature, we then extracted de novo mutational signatures from CMTs using the non-negative matrix factorization (NMF) method (Figure S1C). Two CMT signatures were extracted and compared with COSMIC signatures. De novo CMT signatures A and B showed the best cosine similarities with Signature 5 and Signature 4, 24 followed by 29 and 18 of human cancer, respectively. CMT signature A, showing distinctive mutations of both C to T and T to C transitions, was newly found in CMTs. This signature showed the highest correlation with human cancer Signature 5 (0.78) which has been found in all cancer types and most cancer samples but has gradually decreasing correlations with the others (top, Figure 2D). CMT Signature B, characterized prominently by a C to A substitution followed by a C to T transition (Figure 2D), showed a result consistent with the previous patterns of which correlations were high with COSMIC Signatures 4 and 24. Additionally, Signatures 18 and 29 which have been observed in several human tumors including breast carcinoma were newly added (bottom, Figure 2D). Signature 18 appears directly relevant to breast cancer, but its etiology remains unknown. On the other hand, Signature 29 has been observed only in non-relevant tumors but is also etiologically related to tobacco.

### 2.3. The PIK3CA Gene Is Most Frequently Mutated in CMTs

We determined seven significantly mutated genes (SMGs) that show a significantly higher mutation rate than the background mutation rate in CMT using the Genome MuSic 2. This number is increased to 195 when includes non-coding genes. We then focused on the seven SMGs that included three uncharacterized novel protein coding genes (*ENSCAFG00000020185, ENSCAFG00000029433,* and *ENSCAFG00000029456*) harboring non-synonymous mutations (Materials and Methods; Table S5). Of note, the *PIK3CA* gene was the most recurrently mutated in CMT (45%) followed by *PRMT3* (25%) and then *ENSCAFG00000020185*, *ENSCAFG00000029433*, and *cOR8S14* (20%) (Figure 2E). All somatic mutations identified from the *PIK3CA* gene changed corresponding amino-acid sequences at six different loci (c.1035T > A (p. Asn345Lys), c.1637A > C (p. Gln546Pro), c.1871C > A (p. Thr624Lys), c.3140A > G (p. His1047Arg), c.3172A > T (p. Ile1058Phe) and c.3197C > T (p. Ala1066Val)) of phosphatidylinositol-4,5-bisphosphate 3-kinase catalytic subunit alpha. Importantly, two variants (c.1637A > C and c.3140A > G) in CMTs were very close or exactly matched to *PIK3CA* mutation hotspots in HBC (c.1633G > A and c.3140A > G), where it is known that the 546th and 1047th amino acid changes from Gln to Pro and His to Arg in CMT, respectively [20]. Not as frequent as these two hotpots, c.1035T > A (p. N345K), the pathogenic mutation of *PIK3CA*, was also shared by HBC and CMTs (Table S6) [21].

### 2.4. Integration of Transcriptome Data Reveals the Functional Role of PIK3CA Key Mutation in CMTs

To investigate biological roles of *PIK3CA* mutations in CMTs, we analyzed four pairs of transcriptomes, two with and two without the *PIK3CA* (A3140G) mutation, which is known as a gain-of-function mutation and a mutational hotspot in both HBC and CMTs [22]. Overall, transcriptome sequencing quality was high with >95% of bases with Q30 and a TIN value of >65 (Table S7). A total of 20,438 transcript expressions were detected (FPKM >0.1) and quantile normalized for further analysis. We detected 655 differentially expressed genes (DEGs) between T/N pairs using the modified t-test provided by Cuffdiff.

**Figure 3:**
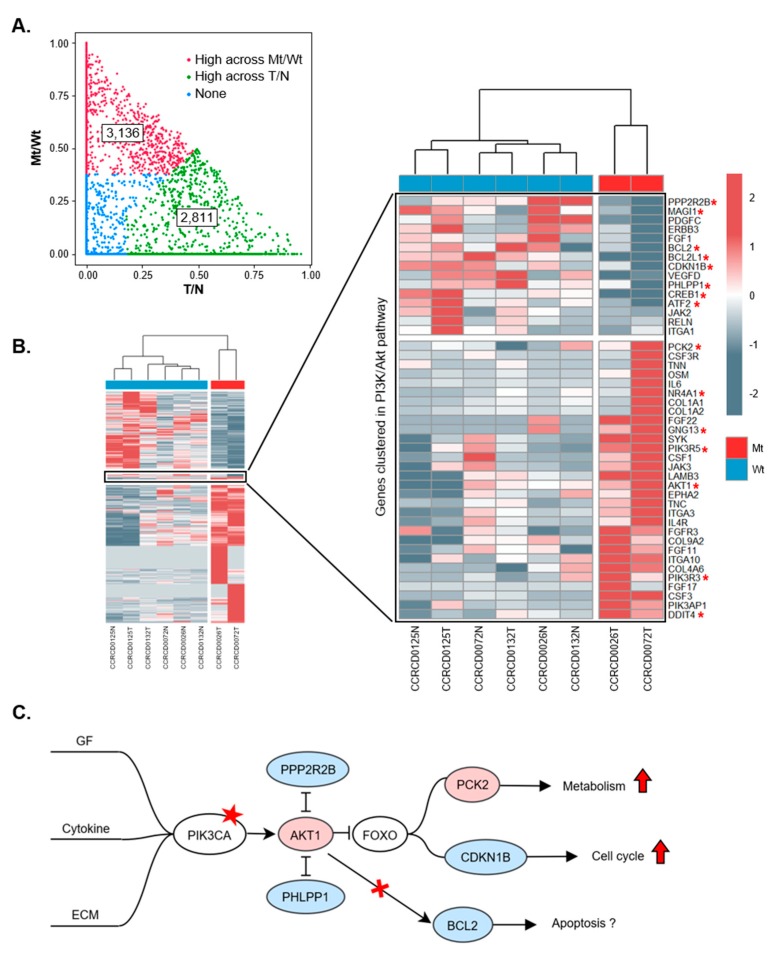
Transcriptional aberration in signal transduction by *PIK3CA* (A3140G) mutations. (**A**) Linear mixed model (LMM) analysis was performed to distinguish between the factor determining T/N and with/without *PIK3CA* (A3140G) mutation (Mt/Wt). Red and green dots indicate genes with top 0.1 quartile in variance and higher than the other fraction across Mt/Wt and T/N, respectively. Blue means no significant differences. (**B**) Heatmap clustering for differentially expressed genes in *PIK3CA* (A3140G) mutation and a zoomed in view of the cluster highlights involvement in the PI3K/Akt signaling pathway. Gene expression was indicated by red (high) and blue (low) color in a z-score scale. * indicates genes downstream of *PIK3CA*. (**C**) The PI3K/Akt signaling pathway with *PIK3CA* H1047R mutation in CMTs. Star indicates *PIK3CA* H1047R mutation. Color in circles represent gene expression (red: increase, blue: decrease). Red arrows mean up-regulation.

Pathway enrichment analysis using the DEGs revealed that ECM–receptor interaction and cytokine–cytokine receptor interaction was highly enriched in CMT. In addition, 26 genes involved in the PI3K/Akt signaling pathway were also determined to have significance (Table S8). To measure the contribution of T/N and *PIK3CA* mutation/wild-type (Mt/Wt) to gene expression variation in CMT, we utilized a linear mixed model (LMM) in lme4 R package. Gene expression was modeled as a function of tumor and the *PIK3CA* mutation considered as random factors for each other [23]. We extracted genes of which the fraction of variance was explained by *PIK3CA* Mt/Wt in the top 0.1 quartile and higher than the other fraction to cluster the samples (Table S9). Of 20,438 expressed genes, 2811 were classified as genes that have a great contribution to variance between T/N states, whereas 3136 genes contributed to variance between Mt/Wt states of *PIK3CA* (A3140G) (Figure 3A). Heatmap clustering clearly visualized that the group of genes differentially expressed under the *PIK3CA* (A3140G) mutation and the 45 genes (30 up-regulated and 15 down-regulated) involved with the PI3K/Akt signaling pathway term in Kyoto Encyclopedia of Genes and Genomes (KEGG: cfa_04151) were clustered in Figure 3B. Of note, *PCK2* encoding phosphoenolpyruvate carboxykinase 2, which has a crucial role that allows cancer to utilize an alternative catabolic pathway, was up-regulated, while *CDKN1B* encoding cyclin-dependent kinase inhibitor 1B, which is known as a cell cycle inhibitor protein, was down-regulated. On the other hand, *BCL2* and *BCL2L1* genes encoding anti-apoptotic proteins which are regulated by PI3K/Akt signaling, were down-regulated in the *PIK3CA* (A3140G) mutation (Figure 3B,C and Figure S2). Moreover, enrichment analysis for a list of 2006 genes which have official gene symbols among 3136 genes was performed using the DAVID software (https://david.ncifcrf.gov/summary.jsp). Interestingly, numerous genes are enriched in the terms of immune related pathways, such as cytokine–cytokine receptor interaction (*p* = 2.1 × 10^−5^) and the TNF signaling pathway (p = 1.4 × 10^−5^) (Table S10).

### 2.5. PIK3CA and Its Mutation Hotspots in CMT Mimics Those in HBC

**Figure 4:**
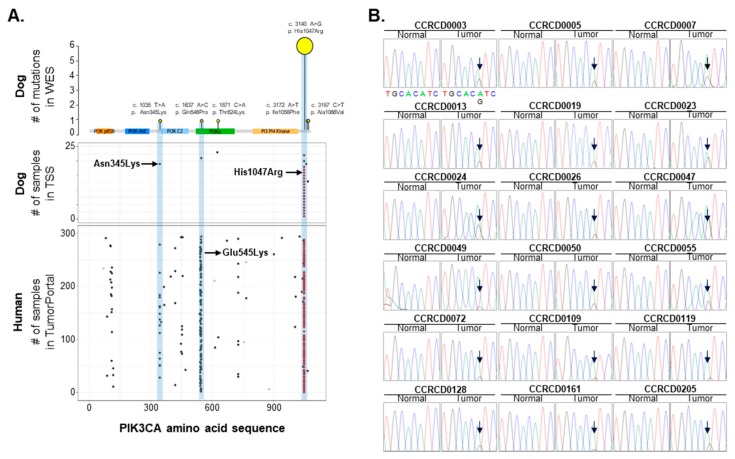
*PIK3CA* mutation hotspot common in CMTs and human breast cancer (HBC). (**A**) *PIK3CA* (H1047R) mutation was common in CMTs and HBC. (**B**) *PIK3CA* (A3140G) mutations changing 1047th amino acid from His to Arg were validated in 18 out of 62 CMT specimens (~29%) by targeted Sanger sequencing (TSS). Arrow indicates mutation hotspot.

The mutationd of three hotspots at the 542nd or 545th and the 1047th amino acids, in exon 9, and exon 20 of *PIK3CA*, respectively, are established in HBC and both mutations are known as gain-of-function mutations resulting in cancer transforming capacity [24]. One of the most interesting findings in this study is that the *PIK3CA* (A3140G) mutation was the most frequent mutation observed in CMTs, indicating a mutational hotspot similar to the mutational hotspot determined in HBC [25,26]. Canine *PIK3CA* has a remarkable 99.8% sequence identity, encoding 99% amino acid identity, with human *PIK3CA* (Figure S3). To investigate whether the mutation hotspots of *PIK3CA* in HBC are conserved in CMTs, we extensively surveyed and validated the mutational loci of *PIK3CA* in CMTs. Eighteen out of 62 CMT specimens (~29%) had the *PIK3CA* (A3140G) mutation. However, other mutations such as Q546P (A1637C), which occurred very close to a second hotspot of E545K (G1633A) in humans, was not recurrent in CMTs as shown through targeted Sanger sequencing (TSS) (Figure 4). Additionally, we surveyed breed tendencies in the occurrence of the *PIK3CA* (A3140G) mutation. A total of 62 CMT validation samples were composed of nine different breeds with a varying number of dogs. The smallest groups, Spitz and Bichon Frise, which included only one and two dogs, respectively, were excluded. Maltese had the biggest sample size encompassing 18 dogs, followed by Schnauzer, Poodle, and Dachshund with eight dogs each (Table S11). Although it is too hard to establish the tendency among breeds due to the small group sizes, Shih-Tzu (50%), Poodle (37.5%), and Maltese (38.9%) showed a higher frequency of the *PIK3CA* c.3140A > G (H1047R) mutation in CMTs than the other four, Schnauzer, Dachshund (25%), Yorkshire Terrier (0%), and Cocker Spaniel (16.7%). However, all these mutation frequencies in CMTs were higher compared to those in HBC (14.3%) [27,28]. This result should be confirmed with a larger sample size and with consideration to CMT breed predispositions. Nevertheless, our result represents the usefulness of the comparative approach in diagnosis and target mutational analysis of both species’ cancers due to the similarity of CMTs to HBC.

### 2.6. CMT-Enriched Somatic Variants Are Capable of Being Used as Novel HBC-Associated Gene Candidates

**Figure 5:**
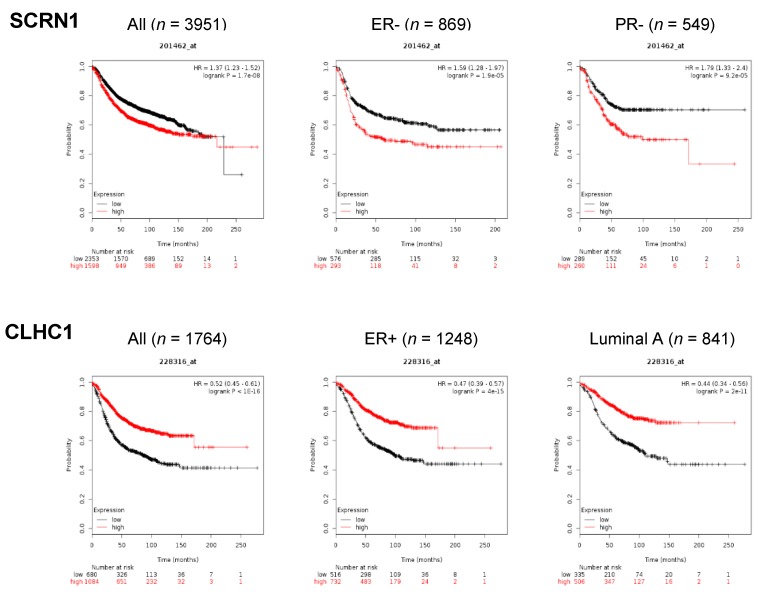
Identification of novel genes associated with breast cancer by KMplot. KMplot analysis for two representative SMGs, *SCRN1* and *CLHC1*, significantly mutated in CMTs. Color indicates gene expression (red: high, black: low). Affymetrix ID 201462_at and 228316_at were used for *SCRN1* and *CLHC1*, respectively. ER (estrogen receptor), PR (progesterone receptor).

To investigate novel HBC-associated genes or mutations, we compared 195 SMGs including mutations in both coding and non-coding regions with a list of 13,134 HBC related genes extracted from the human gene database, GeneCards (https://www.genecards.org), and subsequentially excluded non-coding genes. We then employed a Kaplan–Meier survival plot analysis (http://www.kmplot.com) to assume the HBC-association of mutations in CMTs. Finally, eight SMGs (*TMEM129, STX8, SCRN1, PRM3, ENKD1, CLHC1, CARD6*, and *CALY*) were found to have a distinct association between expression level and overall survival (OS) (Figure 5, Figure S4). Overall, the high expression level of candidate genes tends to have a positive correlation with better OS, except for *SCRN1*. Although *ENKD1* (*p* < 0.05), *CALY* (*p* < 0.05), and *STX8* (*p* < 0.05) showed significant *p*-values, there was no discrepancy in OS between high and low gene expression at 150 months. On the other hand, high gene expression of *CLHC1* (HR = 0.52, *p* < 1 × 10^−16^), *PRM3* (HR = 0.62, *p* = 1.8 × 10^−9^), *TMEM129* (HR = 0.68, *p* = 1.1 × 10^−6^), and *CARD6* (HR = 0.8, *p* < 0.01) presented a significant association with better survival at 150 months. (Figure 5 and Figure S4). Notably, four variants were identified from *CLHC1*; one occurred in the region of the Clathrin H link domain and caused the gain of stop (p. Glu311*) at a position which might be important in gene function, whereas the other three mutations occurred in different intron regions which might be able to modify gene expression regulations (Table S4). Although many mutations have been identified from *CLHC1* in various human cancer types including HBC, their potential roles in cancer are largely unknown and demand further study [29].

### 2.7. Breed Frequency and Enrichment Analysis of Germline Variants May Suggest Genetic Disease Predisposition in Schnauzer and Maltese Dogs

High incidence of certain cancers in several dog breeds are widely recognized by veterinarians and pathologists [30]. Using the whole exome sequencing (WES) data of normal tissues, we investigated fixed or nearly fixed-high frequency alleles in the two breeds, respectively. In particular, since diverse neoplastic conditions, such as melanoma, cutaneous histiocytoma, and trichoepithelioma, have been frequently documented in the Schnauzer breed and epithelial tumors in the Maltese breed, surveying potential breed predispositions to CMTs is important [31,32]. We indeed first surveyed variant alleles existing exclusively in all individual dogs of each breed. Unfortunately, since not all patient dogs are pure in breed, no completely fixed allele (allele frequency (AF):1) was found from either breed.

**Figure 6:**
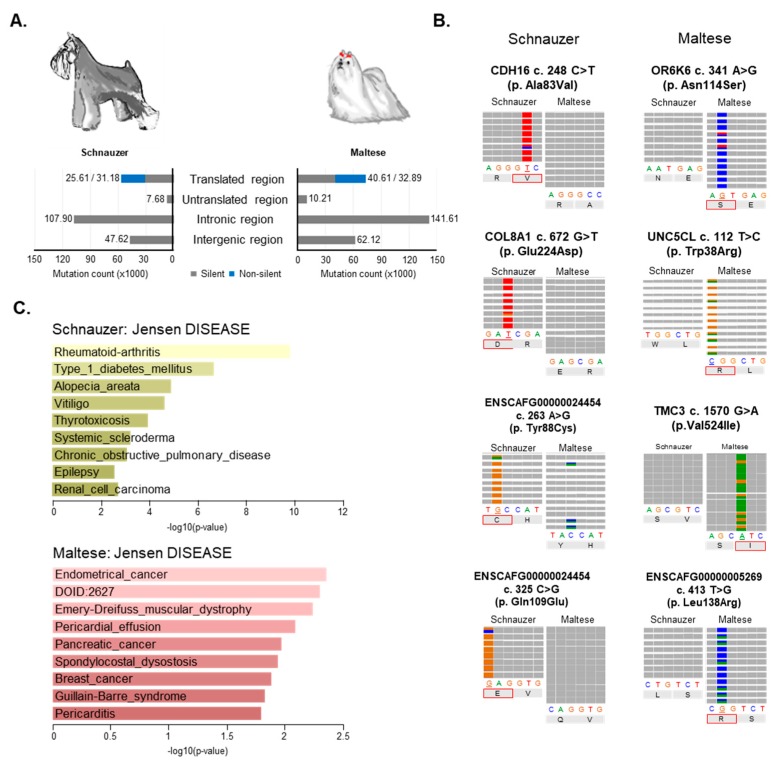
Breed predisposition. (**A**) Distribution of germline mutations in genomic features. Genomic distribution of germline mutations was depicted in each breed and mutations on the translated region were counted based on silent (gray) and non-silent mutations (blue). (**B**) Each breed-enriched germline variant presenting the representative loci with top four average AF in integrative genomics viewer (IGV). The four colors indicated four nucleotides and corresponding amino acid was indicated at missense mutations. (**C**) Jensen DISEASE analysis of breed enriched germline mutations by Enrichr (http://amp.pharm.mssm.edu/Enrichr/).

Approximately, 39% and 48%, respectively, in Schnauzer and Maltese of germline variants on exonic regions were determined as non-silent variants [33]. The largest numbers of variants were captured from intron regions (107,903 (49.3%) and 141,607 (49.1%), respectively, in Schnauzer and Maltese) (Figure 6A). The top four representative germline variants based on AF and exclusively found in each dog breed are depicted in Figure 6B. All four variants depicted in both dog breeds were non-synonymous mutations that cause changes of amino acid sequence (Figure 6B).

In eight Schnauzers, a total of 166 variants (110 genes), including 39 that were non-synonymous, were frequently found in all eight dogs with diverse AFs, ranging from 0.75 through 0.93 (Table S12). Enrichment analysis using Jensen DISEASES, tool to identify the association between gene and diseases using text mining, revealed that these 110 genes are potentially involved in diverse diseases, such as Rheumatoid arthritis (*p* = 1.60 × 10^−10^) and Type 1 diabetes mellitus (*p* = 2.24 × 10^−7^) (Figure 6C, Table S13) [34]. Interestingly, nine genes*, DDR1, BAG6, SLC44A4, EHMT2, FLOT1, HLA-DPB1, LY6G6C, VARS2,* and *MUC21,* were enriched in Type 1 diabetes mellitus which is a very common endocrine disorder in Schnauzers [35]. Notably, of 39 non-synonymous variants on 31 genes, three genes, Cadherin 16 (*CDH16*) and Cadherin 6 (*CDH6*), and Solute Carrier Family 39 Member 7 (*SLC39A7*) have been known to be involved in several carcinogenesis (renal cell carcinoma, *p* < 0.01; estrogen-receptor positive breast cancer and nephrogenic adenoma, *p* < 0.05). In addition, variant alleles of *CDH16* and *COL8A1* genes in Schnauzers might be an important link in understanding the high incidence of renal failure in Miniature Schnauzers, which are considered to have a breed predisposition to renal diseases [36]. On the other hand, 37 germline variants (five non-synonymous) in 23 genes were found with diverse AFs (AF > 0.7) exclusively in the 12 Maltese dogs (Table S14). Gene enrichment analysis in terms of Jensen DISEASES revealed that genes with germline variants (*PLXNA2*, *FAT2,* and *SYNE1*) enriched in the Maltese breed are also enriched in several cancers, such as endometrial cancer (*p* < 0.01), pancreatic cancer (*p* < 0.05), and breast cancer (*p* < 0.05) (Figure 6C, Table S15). Interestingly, three out of the five non-synonymous mutated genes, Transmembrane Channel Like 3 (*TMC3*), Olfactory Receptor Family 6 Subfamily K Member 6 (*OR6K6*), and Unc-5 Family C-Terminal Like (*UNC5CL*), have been reported on in regard to an involvement with various cancer types [37,38].

## 3. Discussion

Many germline and somatic variants are widely considered to be associated with cancers [39,40]. Mutations of several cancer driver genes such as *BRCA*, *PIK3CA*, *ERBB2/ 3,* and *TP53* have frequently been reported in HBC, but rare mutations are still veiled due to the high heterogeneity of humans [41,42]. CMTs have been proposed as a great model for HBC due to the similarity in molecular biology and pathophysiology [7]. Moreover, using CMTs is beneficial to study certain rare types of HBC, such as myoepithelial cell-type HBC, which is very rare (~1% of all HBC) and yet there is a high occurrence of myoepithelial cell-type CMTs (~20%) [43]. However, only a few genome-wide association studies (GWAS) and somatic mutation studies have been performed in dog and dog diseases, including CMTs, and a limited number of genes and variants, such as *CDK5RAP2* and *POLD1*, have been reported [44,45]. In addition, the importance of in-depth variants analysis in non-coding regions, such as introns and intergenic as well as promoter regions, has increased since many recent studies have reported that mutations on non-coding regions also have a strong association with various diseases [46].

One of the most interesting findings in the present study was the high frequency of transversion mutations, which have been demonstrated in smoking-related human lung cancers, represented in CMT mutation signatures [47]. Of note, human cancer mutational Signatures 4 and 29 that are related to tobacco mutagens showed high correlation with CMT signatures as well. It has been proposed that exposure to secondhand smoke can increase a woman’s risk of breast cancer [48]. Altogether, if the high frequency of transversion mutations in CMT is due to the susceptibility of dogs to air pollution, such as smoking and chemicals, further investigation with environmental factors such as smoking habits of the dogs’ guardians will be necessary. For instance, a survey on the residential environments of a CCRCD0009 dog presenting only Signature B will help further understanding (Figure S1). In addition, CMT mutational signatures defined by de novo analysis revealed that CMTs are more likely to be due to environmental factors than hereditary genetic cancer signatures. This allows us to suggest that the comparative study of CMTs and HBC might be helpful in environmental factor-contributing cancer studies.

We also demonstrated that *PIK3CA* mutations are key somatic driver mutations in CMTs just as in HBC [49]. Since 2004, when *PIK3CA* mutations were reported in solid tumors for the first time, numerous studies and trials have been performed and have revealed associations of *PIK3CA* with HBC [50]. In dogs, *PIK3CA* mutation was recently introduced as an actionable mutation in hemangiosarcoma with a low occurrence rate [51]. However, it has not yet been clearly determined whether *PIK3CA* somatic mutations are also involved in the naturally occurring mammary tumors in other species. In our dataset, *PIK3CA* was the most frequently mutated gene in CMTs; we originally discovered through exome sequencing that nine out of 20 (45%) CMTs had *PIK3CA* mutations and also confirmed the *PIK3CA* (A3140G:H1047R) mutation in 18 out of 62 (29%) CMTs of various breeds through targeted sequencing. It is especially important that we found the exact same mutational hotspot at the 1047th amino acids of *PIK3CA* (H1047R) of HBC [50] in CMTs, since canines share a close environment with humans. It remains unclear what mechanism triggers constitutive PI3K signaling [50]. We integrated RNA-seq with WES and revealed that the gain-of-function mutation of *PIK3CA* (A3140G) via H1047R amino acid substitution causing constitutive activation was recurrent in six out of 20 WES and in 18 out of 62 targeted PCRs. Biased tumor occurrence by *PIK3CA* (A3140G) via H1047R, higher in CMTs than in other cancers such as hemangiosarcoma, might be related with the difference of somatic mutation profiles in tumor arising in men and woman [51,52]. Differential regulation of downstream genes in CMTs with the *PIK3CA* (H1047R) mutation, such as up-regulated *PCK2* and down-regulated *CDKN1B*, allows cancer cells to utilize an alternative catabolic pathway. Unexpectedly, *BCL2* and *BCL2L1*, known as genes encoding anti-apoptotic proteins and regulated by PI3K/AKT signaling, were down-regulated in *PIK3CA* (H1047R) mutation. This result should be investigated further with more extensive data required to explain the selective role of the *PIK3CA* (A3140G: H1047R) mutation in CMTs and HBC.

We further identified several SMGs which were recurrently mutated in CMTs but have not been investigated in HBC yet. Kaplan Meier (KM) plot analysis using the list of genes such as *CARD6*, *PRM3*, *TMEM129* and *CLHC1* in HBC revealed that high expression of these genes is significantly associated with better overall survival in overall HBC and HBC subtypes. Particularly, two SMGs, *CLHC1* and *PRM3*, of which expressions have the most significant association with better overall survival in HBC (*p* < 1 × 10^−16^ and *p* = 1.8 × 10^−9^, respectively) may suggest that comparative study using CMT will be useful for better understanding HBC. A future functional study of the mutations on these genes in tumorigenesis and cancer malignancy will be necessary.

Dobson literarily reviewed canine breed susceptibility to various cancers, such as histiocytic sarcoma, melanoma, and mammary tumors [12]. Understanding putative germline variants predisposed to breed enriched diseases, such as tumors, is crucial for a comparative approach using the dog model. We analyzed the germline mutations enriched in each breed to explain the frequent occurrence of neoplastic diseases in each breed. Although this analysis has some limitations such as only two selected breeds with small sample sizes and no finding of completely fixed alleles, numerous SNPs enriched in Schnauzer rather than Maltese and vise-versa were found that related to diverse disease processes, biological processes and molecular functions involved in carcinogenesis. It may yet be meaningful that enriched germline variants of *CDH16*, *CDH6*, and *SLC39A7* in Schnauzer and *PLXNA2*, *FAT2*, and *SYNE1* in Maltese have been known to be involved in several instances of carcinogenesis including estrogen-receptor positive breast cancer and breast cancer (*p* < 0.05). Further investigation in additional dog breeds and larger sample size is necessary to conclude the association of these germline mutations to breed predisposed carcinogenesis.

Although this study still has some limitations, such as (1) the number of cases (twenty) is small compared to the high incidence of mammary tumors in companion dogs and extensive heterogeneity across tumors; (2) this sample cohort includes several variables, such as two different breeds and several CMT subtypes; (3) regulatory mutations may also be important but are not studied here, this study may allow us to better understand both HBC and CMTs and lend new insights into the development of biomarkers.

## 4. Materials and Methods

### 4.1. Specimens

This study was reviewed and approved by the Seoul National University Institutional Animal Care and Use Committee (IACUC# SNU-170602-1). Twenty dogs from two breeds, Maltese and Schnauzer, diagnosed with canine mammary tumors (CMTs) were enrolled in this study, and CMTs and matching adjacent normal tissues were obtained by excisional surgery. In detail, CMTs were diagnosed by needle biopsy. None of the CMT dogs had been treated with chemotherapy before surgery, and matching normal tissue was obtained from another side of mammary gland region without symptoms. Clinical features, including age, sex, spay, breed, as well as cancer category (benign or malignant), are listed in Table S1.

### 4.2. Genomic DNA Isolation and Total Canine Whole Exome Sequencing

Genomic DNA was isolated from 20 pairs of CMTs and adjacent normal tissues using the DNeasy blood and tissue kit (QIAGEN, Hilden, Germany) according to the manufacturer’s guidelines. Genomic DNA was eluted into 50 µL of buffer. Whole exome libraries were constructed by DNAlink (Seoul, Korea) using the SureSelect Canine All Exon Target Enrichment System (Agilent Technologies, Santa Clara, CA, USA) and paired-end Illumina sequencing was run on the Hiseq2500 platform (Illumina, San Diego, CA, USA). FastQC was used to assess the quality of paired-end sequences and the percentage of bases with quality score above 30, which is considered high quality data, in each sample was ~90% (Table S2).

### 4.3. Variant Calling and Annotation

The reads were aligned to CanFam3.1 by the Burrows–Wheeler Aligner MEM algorithm. Initial alignments were refined by local realignment and base quality recalibration using GATK tools (ver. 3.5) Variant calling was processed by two tools: Varscan2 [18] for germline and Strelka2 [53] for somatic mutations, and functional annotation was performed by the SnpEff tool [54]. All parameters were used as default (Figure 1).

### 4.4. Cancer Mutation Signature Extraction

CMT enriched-mutational signatures were extracted from the mutation count matrix by non-negative matrix factorization (NMF) and compared to the HBC mutational signature in the mutational patterns of R Package [55].

### 4.5. Significantly Mutated Genes in T/N

To determine effective mutations and putative driver genes in CMTs, we employed the Genome MuSiC 2 program with the merge-concurrent-muts option [56]. The variants functionally annotated by SnpEff v4.2 were subjected to evaluate the significance of mutations by gene. Significantly mutated genes (SMGs) were identified with a false discovery rate (FDR) threshold of 0.1 using the convolution test (CT).

### 4.6. RNA-Seq Data Integration

Four pairs of CMTs and adjacent normal tissues (T/N) were processed for RNA sequencing. RNA was isolated by RNeasy plus mini kit (Qiagen) according to the manufacturer’s instructions. Initial RNA quality was assessed by analysis of 18S and 28S rRNA band integrity on an RNA 6000 Nano Kit (part # 5067-1511) using an Agilent Bioanalyzer (Agilent Technologies). The transcript integrity number was also checked after sequencing. The sequencing library was constructed using TruSeq Stranded Total RNA sample preparation kit (RS-122-9007), and next generation sequencing was performed on Hiseq2500 platform, by Theragen Inc (Seoul, Korea). Total RNA-seq was done at a length of 100 bp via the paired-end method. After the filtration of low quality reads and adaptor trimming with FastQC and trim_galore, sequences were mapped and annotated to the CanFam3.1 reference genome using Hisat2 (ver. 2.1.0) with the cufflink option for further analysis. Differentially expressed genes were identified using Cuffquant (ver. 2.2.1). To measure the contribution of the PIK3CA (A3140G) mutation in gene expression, the lme4 of R package was employed for the linear mixed model (LMM) [27].

### 4.7. Gene Ontology and Pathway Enrichment Analysis

A list of genes with germline and somatic variants was submitted to Enrichr (http://amp.pharm.mssm.edu/Enrichr/) for the enrichment analysis of pathway and gene ontology. Terms enriched in each analysis were sorted by p-value.

### 4.8. Validations 

Targeted Sanger sequencing (TSS) was performed to amplify two flanked regions, including the mutation hotspots of *PIK3CA* at the 545th and 1047th nucleotide in NC_006616.3. The sequences of the 3140primer set are: 3140F1: CTGGAATGCCAGAACTACAATC and 3140R1: CTGTTCATGGATTGTGCAATTCC, and for the 1637 primer set they are 1637F1: TTCGCCATTTTCTCTTTTTGTAGA and 1637R1: AGGTATGGTAAAACCTGCAAGATA. These primer sets amplify the flanking regions of nucleotide positions 1540-1659 and 3023-3207 harboring two *PIK3CA* mutation hotspots, respectively. Amplicons were sequenced and analyzed by Chromas software (ver. 2.6.6). For the purpose of quality control, TSS was performed by two different companies in some replicated samples. The validation set of CMT specimens are listed in Table S15.

## 5. Conclusions

Taken together, this study provides a comprehensive analysis of WES association with CMTs, its comparison to HBC, and the integrated transcriptome to investigate functional roles of mutations. The results allow us to suggest that (1) the mutational profile in CMTs is similar to HBC related to tobacco carcinogens, (2) *PIK3CA* (A3140G: H1047R) mutation and its signaling pathway is a major driver mutation in CMTs, (3) the comparative approach for HBC using CMTs is very useful to identify novel mutations and genes which might be associated with HBC, and (4) further functional studies will be necessary for the missense mutations on these genes in diverse tumorigenesis and tumor malignancy.

## Supplementary Materials

The following are available online at https://www.mdpi.com/2072-6694/11/12/2006/s1, Figure S1: Mutational signature, Figure S2: PI3K/Akt signaling pathway in the Kyoto Encyclopedia of Genes and Genomes (KEGG) pathway analysis, Figure S3: Amino acid sequence alignment of *PIK3CA* in human and dog, Figure S4: KM plot analysis for SMGs in CMT but have not been studied in HBC, Table S1: Specimen, Table S1: Specimen, Table S2: Statistics of Exome sequencing, Table S3: Total number of germline and somatic mutations per dog, Table S4: Somatic mutations, Table S5: Significantly mutated genes, Table S6: *PIK3CA* mutations in CMTs, Table S7: Statistics for RNA sequencing, Table S8: Differentially expressed gene(DEG) KEGG pathway anaylsis, Table S9: A list of genes contributing to T/N or *PIK3CA* Mt/Wt in LMM analysis, Table S10: KEGG pathway analysis of DEG by *PIK3CA* mutation, Table S11: Specimen information used for validation, Table S12: Schnauzer enriched germline mutations, Table S13: Jensen DISEASE analysis of schnauzer enriched germline mutations, Table S14: Maltese enriched germline mutations, Table S15: Jensen DISEASE analysis of maltese enriched germline mutations. The sequence data from this study has been submitted to the NCBI Sequence Read Archive (SRA accession number: SRR9911342-SRR9911381; http://www.ncbi.nlm.nih.gov/sra/).
