# Supplementary material for: Somatic Mutation of PIK3CA (H1047R) Is a Common Driver Mutation Hotspot in Canine Mammary Tumors as Well as Human Breast Cancers
Source: Cancers (Basel). 2019 Dec 12;11(12):2006. doi: 10.3390/cancers11122006 (PMC6966585; doi:10.3390/cancers11122006)
Supplement: Supplementary file 1 [file cancers-11-02006-s001.zip › Supplementary figures.pptx]

## Slide 1
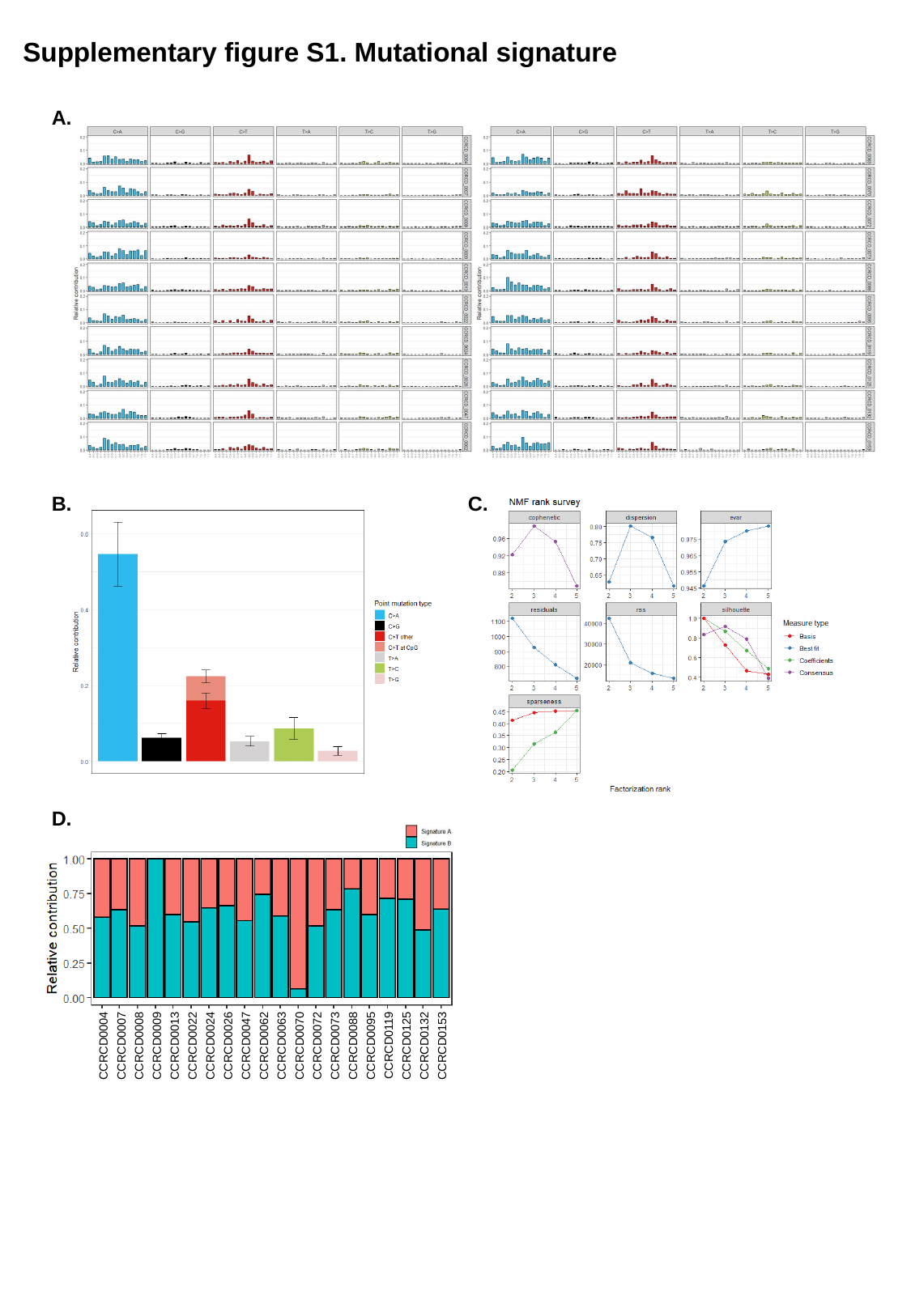

Supplementary figure S1. Mutational signature
A.
B.
C.
D.
CCRCD0004
CCRCD0007
CCRCD0008
CCRCD0009
CCRCD0013
CCRCD0022
CCRCD0024
CCRCD0026
CCRCD0047
CCRCD0062
CCRCD0063
CCRCD0070
CCRCD0072
CCRCD0073
CCRCD0088
CCRCD0095
CCRCD0119
CCRCD0125
CCRCD0132
CCRCD0153

## Slide 2
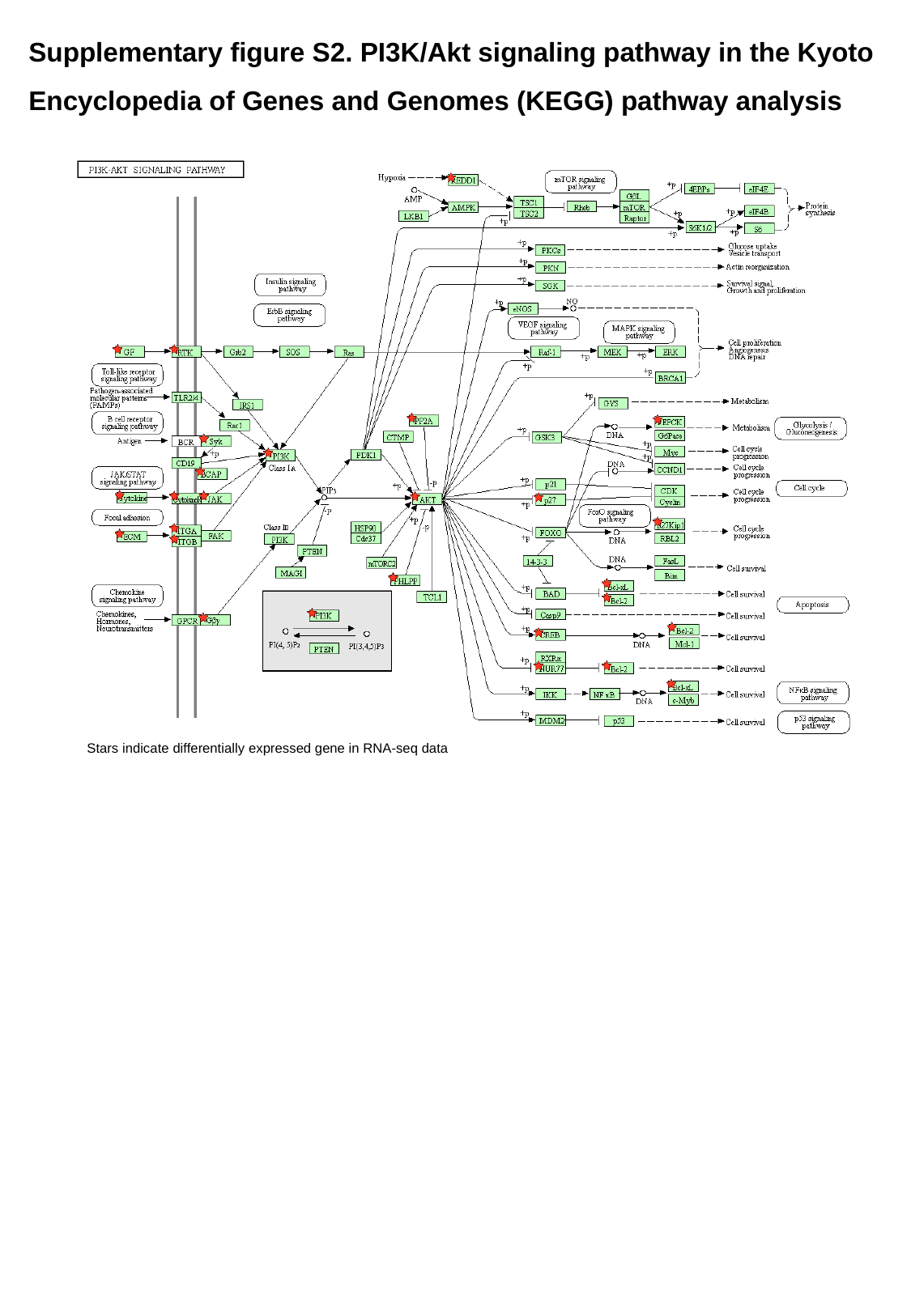

Supplementary figure S2. PI3K/Akt signaling pathway in the Kyoto Encyclopedia of Genes and Genomes (KEGG) pathway analysis
Stars indicate differentially expressed gene in RNA-seq data

## Slide 3
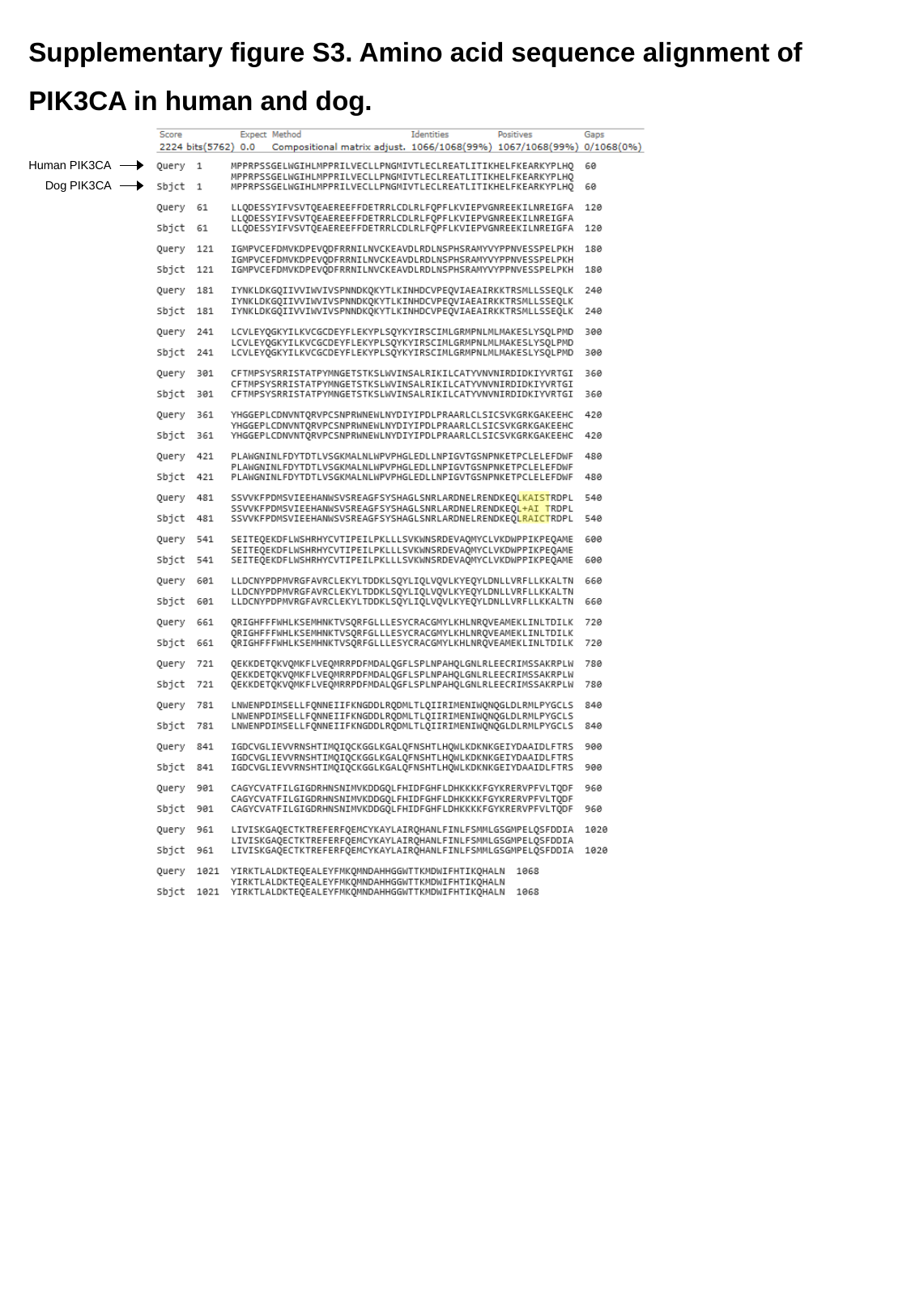

Supplementary figure S3. Amino acid sequence alignment of PIK3CA in human and dog.
Human PIK3CA
Dog PIK3CA

## Slide 4
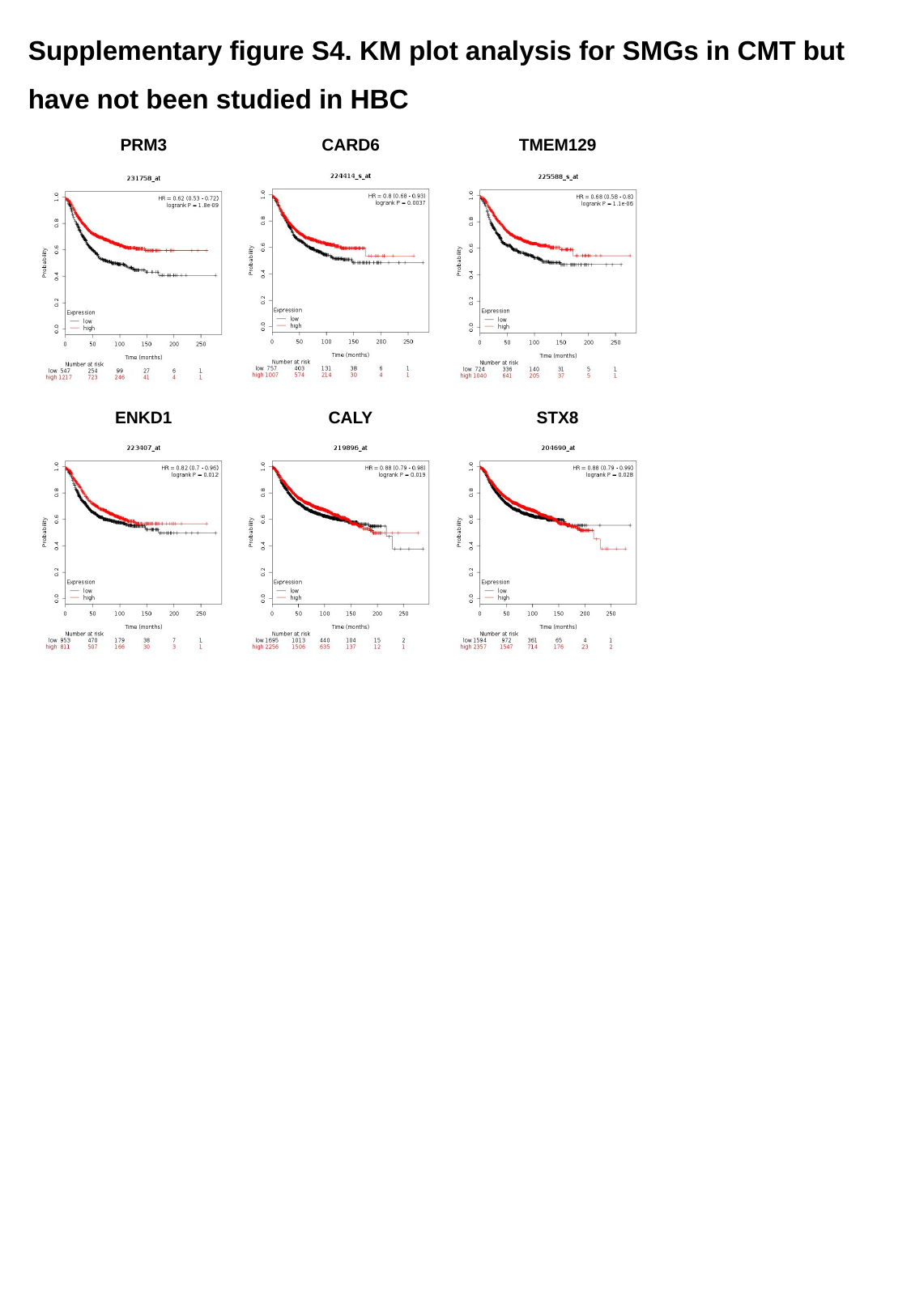

Supplementary figure S4. KM plot analysis for SMGs in CMT but have not been studied in HBC
PRM3
CARD6
TMEM129
ENKD1
CALY
STX8
